# BabyGel pilot: a pilot cluster randomised trial of the provision of alcohol handgel to postpartum mothers to prevent neonatal and young infant infection-related morbidity in the community

**DOI:** 10.1186/s40814-019-0432-7

**Published:** 2019-03-26

**Authors:** J. Ditai, J. Abeso, N. M. Odeke, N. Mobbs, J. Dusabe-Richards, M. Mudoola, E. D. Carrol, P. Olupot-Olupot, J. Storr, A. Medina-Lara, M. Gladstone, E. B. Faragher, A. D. Weeks

**Affiliations:** 1 Sanyu Africa Research Institute (SAfRI), Mbale Regional Referral Hospital, Pallisa-Kumi Road Junction, P.O Box 2190, Mbale, Uganda; 2 Sanyu Research Unit, Department of Women’s and Children’s Health, Liverpool Women’s Hospital, University of Liverpool, Crown Street, Liverpool, L8 7SS UK; 3 Department of Paediatrics and Child Health, Mbale Regional Referral Hospital, Mbale, Uganda; 4 Busitema University Faculty of Health Sciences, P.O Box 1460, Mbale, Uganda; 5 Tropical Clinical Trials Unit, Liverpool School of Tropical Medicine, Pembroke Place, Liverpool, L3 5QA UK; 6 Department of Clinical Infection, Microbiology and Immunology, Institute of Infection and Global Health, University of Liverpool, 8 West Derby Street, Liverpool, L69 7BE UK; 7 S3 Global, London, UK; 8 Health Economics Group, University of Exeter, Exeter, UK

**Keywords:** Infant sepsis, Infection, Morbidity alcohol-based hand rub, Mothers, Hand hygiene, BabyGel, Pilot trial

## Abstract

**Background:**

Alcohol-based hand rub (ABHR) is widely used in both health and social facilities to prevent infection, but it is not known whether supplying it for regular perinatal use can prevent newborn sepsis in African rural homes. Our study piloted a cluster randomised trial of providing ABHR to postpartum mothers to prevent neonatal infection-related morbidity in the communities.

**Methods:**

We conducted a pilot parallel cluster randomised controlled trial across ten villages (clusters) in rural Eastern Uganda. Pregnant women of over 34 weeks’ gestation were recruited over a period of 3 months. Both clusters received the standard of care of antenatal health education, Maama Kit, and clinic appointments. In addition, women in the intervention villages received ABHR, instructions on ABHR use, a poster on the ‘three moments of hand hygiene’, and training. We followed up each mother-baby pair for 3 months after birth and measured rates of consent, recruitment, and follow-up (our target rate was more than 80%). Other measures included ABHR use (the acceptable use was more than four times a day) and its mode of distribution (village health workers (VHWs) or pharmacy), acceptability of study protocol and electronic data capture, and the use of WHO Integrated Management of Childhood Illness (IMCI) tool to screen for newborn infection.

**Results:**

We selected 36% (10/28) of villages for randomisation to either intervention or control. Over 12 weeks, 176 pregnant women were screened and 58.5% (103/176) were eligible. All, 100% (103/103), eligible women gave consent and were enrolled into the trial (55 intervention and 48 control). After birth, 94.5% (52/55) of mothers in the intervention and 100% (48/48) of mothers in the control villages were followed up within 72 h. Most, 90.9% (50/55), of the mothers in the intervention villages (96.2% of live births) and 95.8% (46/48) of mothers in the control villages (95.9% of live births) were followed up at 3 months. In intervention villages, the average hand rub use was 6.6 times per day. VHWs accounted for all ABHR stock, compared to the pharmacy that could not account for 5 l of ABHR. The screening tool was positive for infection among a third of babies, i.e. 29.2% (14/48) in the intervention villages versus 31.4% (16/51) in the control villages.

VHWs completed the first four questions of IMCI screening tool with ease and accuracy. There were no adverse reactions with the ABHR.

**Conclusion:**

It is feasible to conduct a cluster-randomised controlled trial (cRCT) of the provision of ABHR to postpartum mothers to prevent neonatal infection-related morbidity in the community in resource-poor settings. Our results indicate that home recruitment promotes excellent follow-up and retention of participants in community trials. The intervention was safe. This pilot study informed the substantial changes necessary in the larger cRCT, including a change in the primary outcome to a composite outcome considering multiple methods of infection detection. A large BabyGel cluster randomised controlled trial is now required.

**Trial registration:**

ISRCTN67852437, registered March 02, 2015

**Trial funding:**

Medical Research Council/WellcomeTrust/DfID (Global Health Trials Scheme)

## Background

Globally, 45% of deaths in children under 5 years occur in the neonatal period [1–3] with nearly 90% occurring in sub-Saharan Africa and South Asia [4–6]. In Uganda, the neonatal mortality rate is 27 deaths per 1000 live births and has not changed for the past decade while the post-neonatal mortality rate is 16 deaths per 1000 live births [7]. In Eastern Uganda, neonatal mortality is higher than the country average at 34 per 1000 live births, [7–9], with 77% presenting with infection-related symptoms [8].

Though pneumonia, diarrhoeal diseases, and sepsis are the leading infectious causes of deaths in children under 5 years annually [1, 10], the neonatal cause-of-death distribution differs between the early (0–6 days of life) and late (7–28 days of life) periods and varies with neonatal mortality rate level. Preterm birth (40.8%) and intrapartum complications (27.0%) account for most early neonatal deaths in all regions of the world while infections cause nearly half of late neonatal deaths [2], the target for this study. In Uganda, 33% of newborns present with a fever, 9% with symptoms of acute respiratory infections, and 20% experience diarrhoea [7]. Most newborn infections and deaths occur in the community and are frequently unreported to the health sector [11].

Evidence shows that these infant infections are diseases of poverty, associated with poor home environments, remoteness, hunger, undernutrition, and lack of access to essential services [4, 5, 12]. Some infections are transmitted directly from the mother’s genital tract at the time of birth, including streptococci, staphylococci, and *Escherichia coli* [13], while most infections are transmitted from toilets, animals, gardens, or other unclean areas through carers’ hands [14], resulting in neonatal tetanus, skin infections, pneumonia, diarrhoea, or septicaemia. The baby’s umbilical stump is a particular risk especially with traditional practices that increase chances of cord infection, such as the umbilical application of baby powder, soil, or manure [15].

Handwashing with soap is a simple and sustainable measure that results in a large reduction in hand contamination, even when used with unclean water [16–18]. Birth attendant and maternal handwashing have been associated with reductions in neonatal mortality [19]. An estimated 40% reduction in neonatal sepsis deaths relate to newborn care practices at home [20]. A systematic review concluded that evidence for the effect of clean birth and postnatal newborn care practices on neonatal mortality was of low quality [20]. The importance of handwashing in preventing infection-related deaths has led WHO to develop guidelines for hand hygiene both within health care settings and in the community [21–24]. However, studies show widespread non-adherence to the household guidelines, often due to lack of water and or washing facilities. Globally, less than 20% wash their hands after defecation [25] compared to the only 10% in Uganda [26]. Uganda thus ranks among the ten countries with the poorest handwashing behaviour, and access to water is not guaranteed for many regions in the country including Mbale.

An alternative could, therefore, be alcohol-based hand rub (ABHR) which is produced locally in Uganda from sugar cane. It costs only US$0.025 (£0.02) per cleansing and is active against a broad range of Gram-positive and Gram-negative aerobic bacteria, fungi, and enveloped and non-enveloped viruses [14, 27] known to cause diarrhoea and lower respiratory tract infections in early childhood. ABHR requires no infrastructure and can be easily distributed to any recruited Ugandan household. It is also quicker than handwashing with soap and has been suggested to increase compliance [28, 29]. It is therefore rapidly scalable, either by including it with birthing kits or through its provision at the time of birth.

Although ABHR was added to the 19th WHO Model List of Essential Medicines, in support for hand hygiene [14, 30], there is insufficient evidence for the prevention of infections in early infancy. No policies as yet recommend the use of ABHR for routine community postnatal prevention of newborn infections. In the BabyGel study, we hypothesise that the addition of ABHR to birthing kits would enable mothers to provide effective hand hygiene for the first three postnatal months and lower the risk of young infant infections.

This study was undertaken to pilot a cluster randomised trial design for the provision of ABHR antenatally for use by postpartum mothers and other baby carers in the prevention of infection-related neonatal morbidity and mortality in these rural communities. The feasibility aims were as follows:

1. To assess whether village leaders and pregnant women are willing to participate in the study (criteria: a participation rate of at least 80%)
2. To test the integrity, feasibility, and acceptability of the study protocol including questionnaires, information sheets, data management systems, and methods for the identification of community neonatal infections (iterative assessment and refinement of processes)
3. To evaluate the use of the WHO IMCI criteria for a community screening of possible infection as a primary outcome (criteria: assessment of the completed IMCI screening tool).
4. To determine the locally appropriate mechanism for the distribution of ABHR through a comparison of the relative benefits of pharmacy-based and VHW-based distribution mechanisms (an iterative assessment)
5. To assess whether participants use ABHR once recruited into the BabyGel trial (acceptable criteria: mean use of at least four times/day)
6. To assess the level of contamination between intervention and control clusters (acceptable criteria: mean alcohol hand rub use of more than once a day in control clusters).
7. To estimate the intracluster correlation coefficient to inform the sample size calculation for a large cluster randomised controlled trial

Other published findings from the BabyGel pilot trial include the acceptability of ABHR for community use [31], the optimising informed consent for trial participation in Uganda [32], and the newborn moments of community hand hygiene [33].

## Methods

### Study design

In this feasibility study, we piloted an open two-arm parallel cluster randomised controlled trial of the provision of ABHR to postpartum mothers to prevent neonatal infection-related morbidity in the home. We used the definitions and methodology for feasibility and pilot studies as recommended in the ‘CONSORT 2010 statement: extension to randomised pilot and feasibility trials’ [34]. The pilot trial was locally approved by the Mbale Regional Hospital Institutional Review Committee (REIRC IN–COM 011/2015), registered with the Uganda National Council for Science and Technology, and prospectively registered (ISRCTN67852437).

### Criteria for site selection

The study village was selected if it had recorded at least ten births within the past 3 months and had one or more active VHWs and the village leaders committed to study participation and implementation.

Meanwhile, the health centres were included if they had participated previously in community trials or research.

### Study setting

**Fig. 1:**
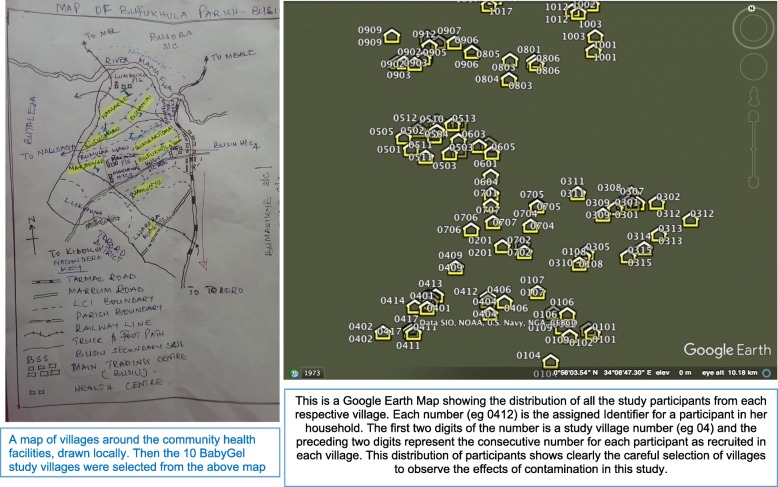
The village map showing the villages drawn and the distribution of participants in each village for the BabyGel pilot trial. A map of villages around the community health facilities drawn locally. The 10 BabyGel study villages were selected from the above map. There is also a Google Earth map showing the distribution of all the study participants from each respective village. Each number (e.g. 0412) is the assigned identifier for a participant in her household. The first two digits of the number is a study village number (e.g. 04) and the preceding two digits represent the consecutive number for each participant as recruited in each village. The first five (01 to 05) were intervention while the last five (06 to 10) were control villages. This distribution of participants shows clearly the careful selection of villages to observe the effects of contamination in this study

**Fig. 2:**
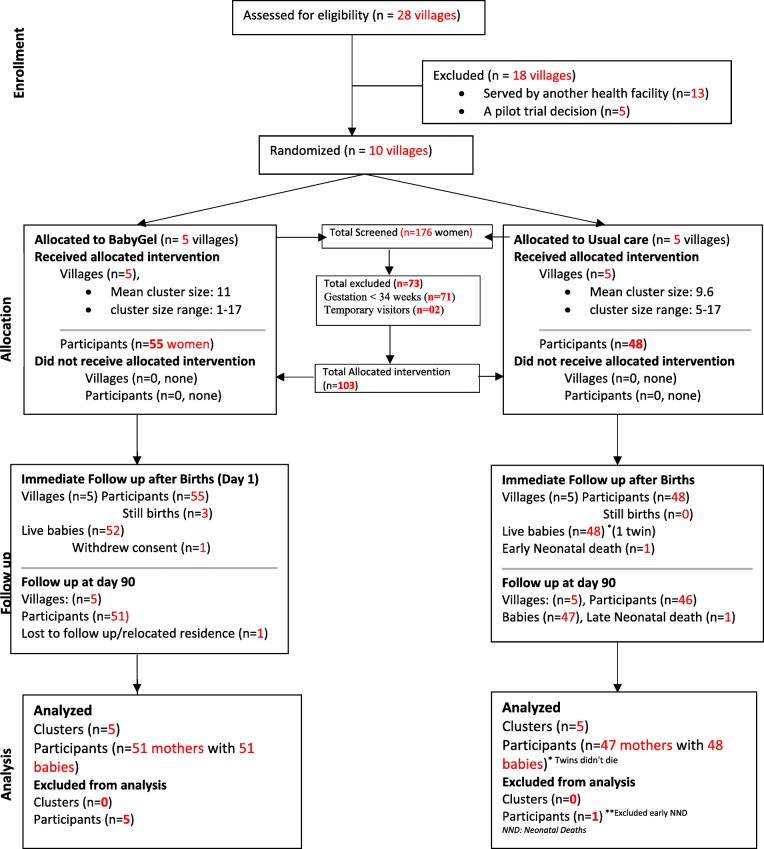
CONSORT diagram showing the flow of participants through each stage of the BabyGel pilot cluster randomised trial. This CONSORT flow chart illustrates the screening and randomisation of clusters and the flow of participants in the study

The study was conducted in ten clusters (villages) around two community health centres (health centre three (HCIII) and health centre four (HCIV)). The HCIII was surrounded by three control villages, while the HCIV was surrounded by five intervention and three control villages. The clusters were rural villages located in Mbale region, Eastern Uganda. A map of the villages surrounding the targeted community health centres in Mbale district was reviewed by the research team in collaboration with the village health team workers (VHWs) (Fig. 1*)*. Ten villages were selected from a map of 28 villages (Fig. 2) if they were within the catchment area of the participating community health centres, were not served by another community health centre, and had a village health worker.

### Pilot randomisation

The ten villages were assigned to either intervention or control by simple random sampling in a central office in Mbale in a 1:1 ratio by a person who did not draw the map. This represented a variety of distances of villages from each other, from market areas, from the health centres, and from the control villages, thus allowing contamination to be effectively assessed. It was not possible to blind women, health workers, local council leaders, or researchers, due to the nature of the intervention.

### Cluster consent

After allocation, researchers met the local council leader(s) of each village to seek consent for participation of their village in the pilot trial and the pilot trial allocation.

### Study timeline

Villages were selected in November 2014, 9 months before recruitment of the first participant for appropriate trial preparation purposes. The recruitment of participants into the trial was time bound. The research midwives recruited eligible women for a period of 12 weeks between August 2015 and November 2015, while each individual woman together with her baby was followed up in her home by research midwives until 12 weeks after childbirth. The overall pilot trial follow-up of participants continued until May 2016 when the last mother-baby pair had their final study assessments at 12 weeks (90 days) after birth.

### Participants’ selection criteria

Pregnant women were eligible for the study if they had a pregnancy with an estimated gestation of over 34 weeks during the recruitment period and were resident in the participating villages. The exclusion criteria included temporary visitors (defined as any pregnant mother found as a visitor in the home and will not stay afterward). Other clinical criteria (e.g. malaria in pregnancy, previous caesarean section) were not used as exclusion criteria in this pilot study due to the nature of the intervention.

### Participant recruitment

Each village health worker (VHW) identified pregnant women from his/her village by direct contact or review of the village-specific monthly pregnancy and birth register. Also, midwives identified pregnant women turning up for their antenatal clinics at the two participating health facilities from the list of study villages. The VHWs or facility midwives then notified a member of the research team about the potential participant. The research team then visited the woman’s home to confirm eligibility, obtain informed consent, and conduct detailed study-specific assessments.

The research midwife screened each woman for eligibility and established the gestational age using a gestational wheel and the self-reported last normal menstrual period (LNMP) [35]. The research team recruited eligible women into the pilot trial for a period of 12 weeks. They provided informed consent for trial participation and follow-up. Those who declined were given the Maama Kit, and their data was not included. The Maama Kit was devised by the Ministry of Health in Uganda to ensure childbirth is conducted in a clean environment. The Maama Kit consists of basic supplies, i.e. sterile gloves, plastic sheets, cord ligature, razor blades, tetracycline, cotton, gauze, soap, and sanitary pads [36].

### Interventions: BabyGel intervention villages

Pregnant women in intervention villages were provided with ABHR (Alsoft V, Saraya East Africa Co. Ltd.) at recruitment. The recruiting research midwives provided the ABHR free of charge to each woman in a 1-l bottle for use while at home, along with a refillable 100-ml bottle for use while travelling.

**Fig. 3:**
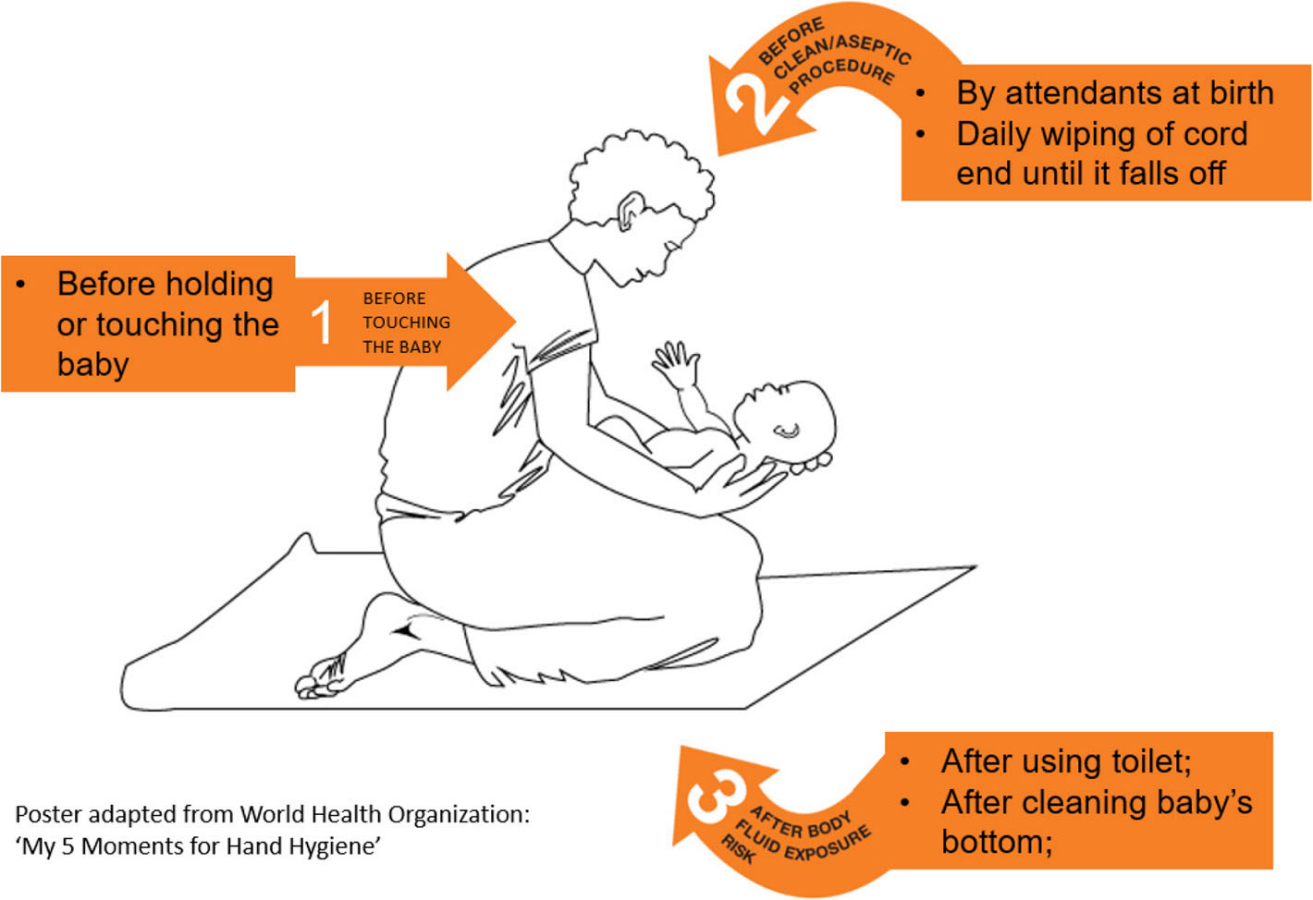
The ‘three moments for community neonatal hand hygiene’ poster developed for the BabyGel pilot trial. This shows an illustrative and diagrammatic representation of the key moments of hand hygiene for newborns in the community

The recruiting midwives trained each woman in the intervention villages on the use of ABHR, the basic hand rub steps, and the ‘three moments for community neonatal hand hygiene’ (Fig. 3), developed by the study team for the pilot trial [33]. This was adopted from the WHO ‘5 Moments for Hand Hygiene’ [14]. The three moments for community neonatal hand hygiene instructions were printed on a poster with a pictorial illustration, which was given to the participants as instructions to display in a visible area and follow in their homes. The poster was available in both English and the local language (Lumasaba). The instructions on the poster recommended hand rub before touching the baby, before clean or aseptic procedures by birth attendants, and daily wiping of cord end with the ABHR three times a day until it falls off. It also included, after any body fluid exposure risk such as after the mother or carer using the toilet, touching any surfaces and exposure to child faeces. The midwives encouraged women to apply the ABHR based on this policy.

In addition to the training and the poster, the recruiting research midwives provided further instructions to the women. This included women commencing the ABHR at the time of recruitment until 3 months after childbirth, each woman performing a whole body wipe of the baby with ABHR within 4 h of birth [37], and every woman encouraging any carer or family member (including children) to use the ABHR with the ‘three moments’ instructions used within and away from their homes.

The research midwives instructed women from three of the five intervention villages to obtain refills from their VHWs (VHW-based distribution) if the need arose, while women from the other two intervention villages refilled from the pharmacy at the health centre IV (pharmacy-based distribution). This allowed us to compare the two modes of ABHR distribution.

Alongside the above intervention, midwives offered the current standard care practice (described below) as was provided for the control villages.

After follow-up for the first 14 participants after birth within the intervention clusters, we recognised that some mothers reported forgetting to use the ABHR as indicated, while some who had homebirths had not taken their newborns to the health facilities for immunisation. However, in almost every household studied, there were already school-going children present. We hence introduced a system of the ‘expert child’ (a child in each house tasked with the responsibility of reminding carers to use the ABHR) to improve ABHR adherence and remind the mother to notify the research team of any sick baby to strengthen the multifaceted approach of the intervention.

In this pilot trial, we limited our communication to the one-to-one teaching of the participants with the use of the poster. This was a small-scale study in which the behaviour change was limited to the participating women and their immediate families. Mass communication or media strategies were, therefore, not necessary.

### Control villages: current standard care practice

Women in the control villages received the current standard care of Maama Kits for delivery and the usual antenatal education. At the time of recruitment, the recruiting midwives advised the women to deliver at health facilities. The midwives encouraged women to attend postnatal checks and immunisation clinics at 6–24 h, 1–2 weeks, 6 weeks, 10 weeks, and 14 weeks at the nearby health facilities in line with the local guidelines [38]. These visits also included information on the importance of hand hygiene in line with WHO guidelines [21] and promote the usual practice of ‘dry care’ of the umbilical cord [39, 40]. The research midwives discouraged women in the control villages from applying any local substances to the cord including home-made saline but use ‘dry care’, keeping the cord clean and dry.

### Trial follow-up procedures

At the end of baseline data collection, the follow-up study visit dates for day 1 and day 90 were auto-generated from the ODK system on the mobile smartphone, based on the expected date of delivery. These scheduled dates were written on the follow-up cards which were given to mothers. On the actual follow-up dates, the research assistant captured the exact dates for day 1 and day 90 assessment on the follow-up card. This enabled some women to remind or call the research team to confirm if they were approaching day 90. The day 90 follow-up schedule from the electronic system guided the research team when to visit the woman.

**Fig. 4:**
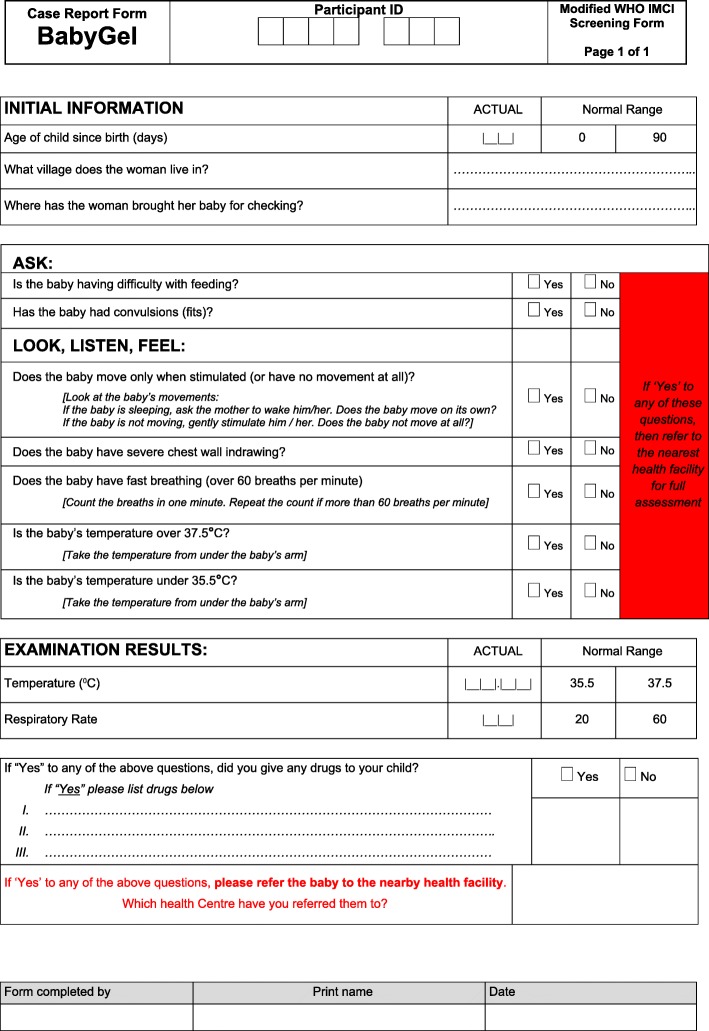
Adapted WHO IMCI screening tool for infection. This tool was administered to newborns at particular encounters as a screening tool for infection

The participant, health centre midwives, or VHWs notified the research team of the birth immediately after birth. The research team also monitored the scheduled expected date of delivery and contacted the participant regularly to see whether they had given birth. The team then visited the woman to collect data as soon as possible after birth. VHWs were encouraged to visit mothers in their homes twice weekly in the first 4 weeks after birth, then once a week thereafter. At each visit, VHWs were asked to complete the WHO IMCI screening tool (Fig. 4). Babies with a positive WHO IMCI screen were referred to the nearest participating health facility for assessment. Meanwhile, mothers with concerns about their babies were also advised to take their babies directly to the health centres where they were screened for infection using the WHO IMCI screening criteria and assessed by a clinician. The research team was notified of any positive WHO IMCI screen by either the VHW or participant or health facility midwife/clinician. The paediatrician based at Mbale hospital was available for consultation for any sick infants presenting at the community health facility and or being admitted. The research team continued to follow up each woman and her baby(ies) until 90 days postnatally. The research team encouraged VHWs to remind the mothers in intervention villages to use the ABHR and return for refills as required. The research assistants also encouraged the use of the ABHR during any follow-up phone calls and on the day 1 follow-up visit.

### Outcome measurements

#### Pilot trial process outcomes

##### Electronic data capture system

The research midwives collected data using the Open Data Kit system (ODK; University of Washington, USA) loaded onto handheld Samsung Galaxy S4 mobile phones. The research team carried these phones into the community for entering data directly from the participants. After completion by a research assistant, three approvers performed quality checks (i.e. the data collected by the research assistant was reviewed and approved by the study coordinator followed by the co-investigator, then data manager). Once approved, the data on the phone automatically synchronised with the server at the Liverpool School of Tropical Medicine either while in the community or upon return to the research office where there was adequate Wi-Fi signal or internet. The research team discussed the structure, content of the questions, format, and font size of the text on the ODK phone after every data collection visit.

Specific versions of questions were developed for data collection at baseline, follow-up at day 1 and day 90 after birth, and any interim visit.

*At baseline visit.* The research midwife collected baseline demographics, hand hygiene exposure, and current obstetric history. The midwife also collected ABHR details including quantity dispensed and how to obtain the refill, for participants from the intervention clusters.

*Day 1 follow-up visit* data included maternal outcomes (antenatal and intrapartum) and neonatal outcomes (age, birth weight, IMCI screening for infection).

*Day 90 follow-up visit* data included neonatal outcomes (medical history, IMCI screening for infection), hand hygiene practices, and ABHR use.

#### Participant-specific outcomes

##### Primary outcome

**Fig. 5:**
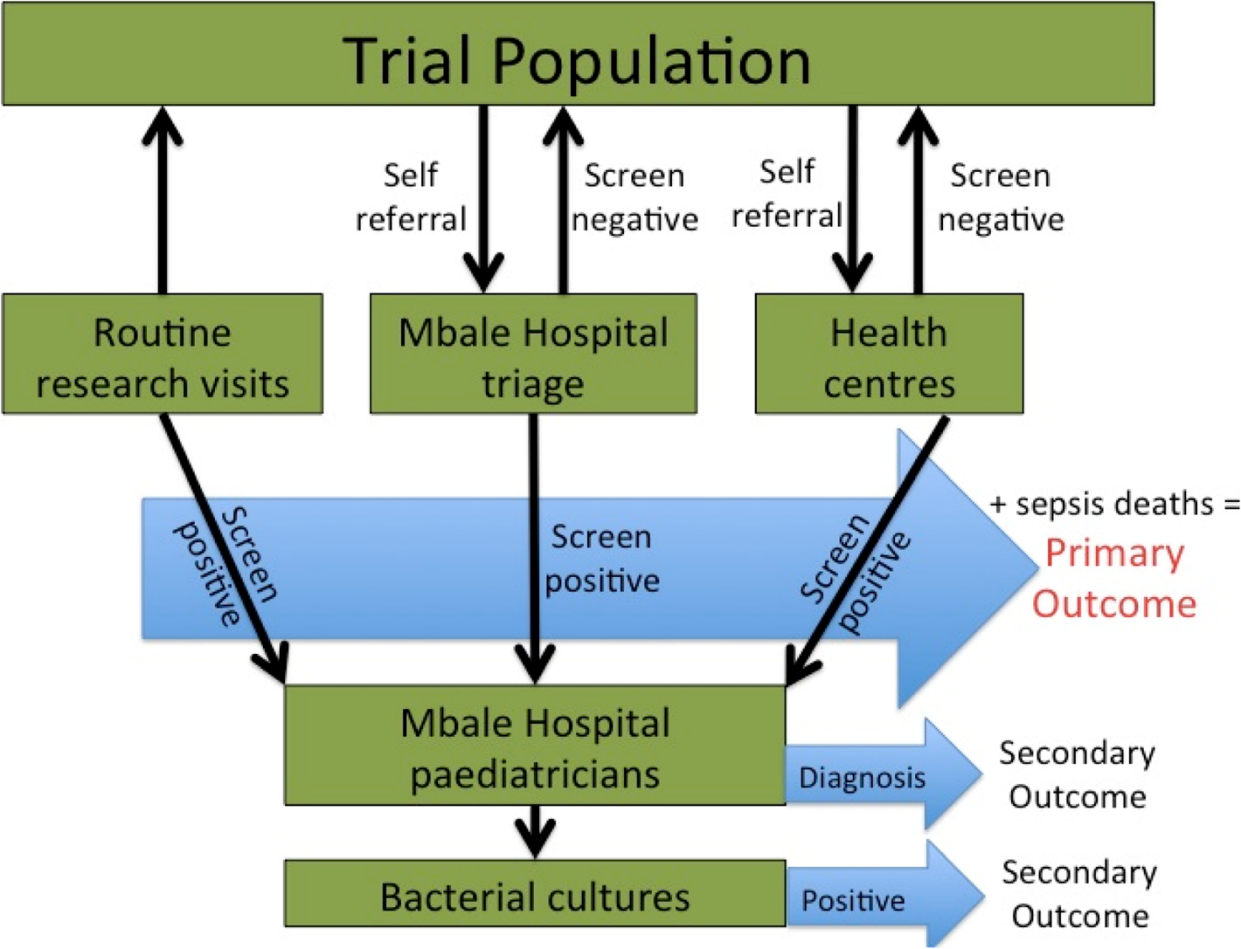
Illustration of the method for the identification of infection-related infant outcomes in the BabyGel pilot trial. Infants participating in the study had the IMCI screening tool administered either at routine research visits or when their mothers brought them to participating hospitals or health centres. The group who screened positive were referred to the paediatric team at Mbale Hospital for clinical care, and along with those who died, composed the primary outcome for the study. Those who screened negative returned to the community. At the hospital, those who had screened positive had both a clinical and bacterial diagnosis performed; these were collected as secondary outcomes

The primary outcome was the number of positive infants on the WHO IMCI screening tool [41, 42] with modifications informed by the YICSSG algorithm [43]. The rates of infection were assessed according to the study-specific neonatal and young infants’ outcomes screening algorithm (Fig. 5). The research midwives assessed newborns in their homes at 1–2 days and 3 months postnatally and applied the WHO IMCI screening tool. The research team encouraged the VHWs to complete the WHO screening tool whenever in contact with the mother-baby pair. Further, the research team asked the staff at health facilities to complete the WHO IMCI screening tool if the baby was presented to the facility without notice of the study team. Any baby screening positive for infection at these visits was referred to Mbale hospital for appropriate management by the paediatrician. The blood sample was collected from all admitted babies in the hospital for malaria blood smear, complete blood count, culture, and sensitivity.

#### Secondary outcomes

##### Infection-related infant mortality

These were sepsis deaths in the first 90 days of life, assessed using verbal autopsy [44]. Infant mortalities at 24 h, 7 days, 4 weeks, and 3 months of life (all cause and infective) were assessed.

##### Maternal quality of life at 3 months after childbirth

The research midwife assessed every woman using the WHO Quality of Life Scale Brief Version (WHOQOL-BREF 1998) [45]. This tool was translated into the local language (Lumasaba) for use and validation in this community.

##### ABHR use

The research midwives recorded the volume of ABHR dispensed to each participant on the e-data capture system at the time of recruitment and at end of study through review of the dispensing and accountability logs. The staff at the health facility pharmacy and VHW in intervention villages maintained dispensing and accountability logs of ABHR distributed to participants. The study team then conducted an inventory of the distributed ABHR to participants from the designated health facility and the VHW’s home. At the last follow-up visit, the research assistant measured the total volume of the ABHR remaining in the bottles to establish the total volume of ABHR used by each participant.

##### Adverse events

The research team collected data on any adverse events related to the ABHR among the newborns. The research team reported any event to the mother or child that required hospitalisation and was life-threatening or a congenital abnormality as a severe adverse event (SAE) irrespective as to whether it is expected or potentially related to the study intervention.

##### Risk of contamination

The research midwives collected data on the ABHR use and knowledge of ABHR, during the follow-up of all mothers in the control villages.

### Community advisory board

A community advisory board (CAB) was set up consisting of a religious leader, a woman representative, a teacher, the local council (LC) 1 chairperson, a VHW, a clinical officer, a motorbike ambulance cyclist, a midwife, the health facility in charge, a doctor, and the Mbale District health officer (chair). Two CAB meetings were held; the first one immediately after the start of the study and the last one at the end of the study. During the meetings, the researchers explored the views of these members about the study and its interventions.

### Sample size

The main objective of this pilot trial was to test the feasibility of providing ABHR antenatally for use by mothers and other baby carers after birth to prevent infection-related neonatal morbidity and mortality in the communities. As this was a pilot trial designed to examine the methodological and procedural uncertainties of a future full-scale trial, we did not undertake a formal sample size calculation. Instead, the data collection period was time limited. With an average of 3 to 4 deliveries per month in the study villages, in a 3-month pilot, therefore, we expected to recruit 90–120 women. This would allow us to assess background infection rates among newborns which we could use for a formal sample size calculation for the full cluster randomised trial (since funded by European and Developing Countries Clinical Trials Partnership (EDCTP)).

### Data analysis

For this pilot trial, the samples were compared as if individually randomised (i.e. ignoring possible clustering within villages). The baseline characteristics of the study mothers are summarised as mean values with their standard deviations and ranges for continuous measures, or as frequency counts and corresponding percentages for categorical measures. Similar statistics are used to summarise all outcome measures. As the study was primarily concerned with determining the feasibility of providing ABHR to prevent infection-related neonatal morbidity, the differences between the two arms are also presented alongside their 95% confidence intervals (calculated using exact binomial methods for categorical measures). No formal statistical comparisons (*p* values) were calculated. The intracluster correlation coefficients (ICC) for possible infant infections among infants at 3 months after birth was also calculated.

## Results

### Acceptability of intervention

#### Cluster consent

All, 100% (10/10), local council leaders of the randomised villages gave informed consent for the participation of their village in the study and study allocation.

#### Participants’ consent

All, 100% (103/103), eligible women gave individual informed consent to participate in the BabyGel pilot trial. However, one woman in the intervention village withdrew her consent from the study after birth, due to pressure from the husband about the ABHR.

#### Community advisory board

All, 100% (12/12), CAB members chaired by the District Health office approved and accepted the pilot trial and the intervention in this area.

### Screening and recruitment outcome

Over a 3-month period from 20 August 2015 to 30 November 2015, 176 pregnant women were screened and 103 met the eligibility from the 10 villages. All, 100% (103/103), eligible women were recruited into the pilot trial. Of the 41.5% (73/176) excluded from the trial, 71 did not meet the inclusion criteria of gestation age less than 34 weeks and 2 others had only visited the homes for a temporary period. Of the 103 participants recruited, 55 were allocated and received the BabyGel intervention, while 48 were allocated and received the usual care. Figure 2 shows the CONSORT flow diagram for the study.

### Demographics

The participants’ demographic characteristics are summarised in Table 1*.* Both types of clusters were evenly matched. The study participants were mostly married (77.1% (37/48) for control compared to 78.2% (43/55) for intervention). About half (45.8% (22/48) for control compared to 56.4% (31/55) for intervention) did not complete primary education level and were either housewives (47.9% (23/48) for control compared to 45.5% (25/55) for intervention) or peasant farmers (37.5% (18/48) for control compared to 36.4% (20/55) for intervention), although those in the intervention villages were more likely to have homes with tap water (either personal or shared) and more likely to have no latrine (Table 1). They generally lived in ‘mud and wattle’ houses with mud floors (47.9% (23/48) for control compared to 45.5% (25/55) for intervention) and corrugated iron roofs (87.5% (42/48) for control compared to 89.1% (49/55) for intervention), and obtained water from a shared tap or borehole (72.9% (35/48) for control compared to 90.9% (50/55) for intervention). Most (91.7% (44/48) for control compared to 80.0% (44/55) for intervention) used non-ventilated pit latrines without the facility for handwashing. Despite this, over 80% of women reported washing their hands more than 50% of times after either urinating or defecating, with about 87% using soap and water (87.5% (42/48) for control compared to 87.3% (48/55) for intervention).

**Table 1:** Demographic/background characteristics (baseline assessment)

		Control group	Intervention group	Both groups combined
Sample size		48	55	103
Age (mean, s.d., range)		24.8 (5.6) [15–37]	25.0 (5.7) [15–38]	24.9 (5.7) [15–38]
Marital status				
Single (*n*, %)		10 (20.8)	12 (21.8)	22 (21.4)
Married (*n*, %)		37 (77.1)	43 (78.2)	80 (77.7)
Widowed (*n*, %)		1 (2.1)	0	1 (1.0)
Highest level of education attained				
No formal education (*n*, %)		2 (4.2)	1 (1.8)	3 (2.9)
Did not complete primary ed. (*n*, %)		22 (45.8)	31 (56.4)	53 (51.5)
Completed primary (PLE) (*n*, %)		16 (33.3)	15 (27.3)	31 (30.1)
Completed ordinary (UCE) (*n*, %)		4 (8.3)	5 (9.1)	9 (8.7)
Completed advanced (*n*, %)		3 (6.3)	2 (3.6)	5 (4.9)
Completed tertiary (*n*, %)		1 (2.1)	1 (1.8)	2 (1.9)
Primary occupation				
Unemployed (*n*, %)		6 (12.5)	3 (5.5)	9 (8.7)
Housewife (*n*, %)		23 (47.9)	25 (45.5)	48 (46.6)
Student (*n*, %)		0	3 (5.5)	3 (2.9)
Peasant farmer (*n*, %)		18 (37.5)	20 (36.4)	38 (36.9)
Businesswoman (*n*, %)		0	2 (3.6)	2 (1.9)
Professional (*n*, %)		1 (2.1)	0	1 (1.0)
Other (*n*, %)		0	2 (3.6) (hotel management, teacher)	2 (1.9)
House roof type				
Iron sheet (*n*, %)		39 (81.3)	52 (94.5)	91 (88.3)
Grass thatched (*n*, %)		9 (18.8)	3 (5.5)	12 (11.7)
House floor type (more than one response possible)				
Mud (*n*, %)		42 (87.5)	49 (89.1)	91 (88.3)
Brick (*n*, %)		10 (20.8)	11 (20.0)	21 (20.4)
Cement/stone/tile (*n*, %)		5 (10.4)	6 (10.9)	11 (10.7)
Other (*n*, %)		1 (2.1) (bricks and mud)	0	1 (1.0)
The main water source for home				
Open water well (*n*, %)		12 (25.0)	1 (1.8)	13 (12.6)
Piped and tapped to home (*n*, %)		1 (2.1)	4 (7.3)	5 (4.9)
Shared tap/borehole (*n*, %)		35 (72.9)	50 (90.9)	85 (82.5)
Type of latrine used in home				
No latrine (use bushes) (*n*, %)		3 (6.3)	8 (14.5)	11 (10.7)
Non-ventilated pit (*n*, %)		44 (91.7)	44 (80.0)	88 (85.4)
Ventilated improved pit (VIP) (*n*, %)		1 (2.1)	3 (5.5)	4 (3.9)
If latrine, type of handwashing facility				
No facility (*n*, %)		32 (71.1)	38 (80.9)	70 (76.1)
Near latrine (*n*, %)		13 (28.9)	4 (8.5)	17 (18.5)
Away from latrine (*n*, %)		0	5 (10.6)	5 (5.4)
Animals/poultry reared/kept (*n*, %)		45 (93.8)	51 (92.7)	96 (93.2)
Cows and goats (*n*, %)		35 (77.8)	37 (72.5)	72 (75.0)
Poultry (*n*, %)		41 (91.1)	49 (96.1)	90 (93.8)
Other (*n*, %)		12 (26.7)	11 (21.6)	23 (24.0)
Pigs (*n*, %)		11 (24.4)	11 (21.6)	22 (22.9)
Ducks (*n*, %)		0	1 (2.0)	1 (1.0)
Rabbits (*n*, %)		1 (2.2)	0	1 (1.0)
Times (out of 10) hands washed when				
Urinating (median[range])		5 [0–10]	4 [0–10]	5 [0–10]
0 (*n*, %)		8 (16.7)	9 (16.4)	17 (16.5)
1–4 (*n*, %)		13 (27.1)	19 (34.5)	32 (31.1)
5–9 (*n*, %)		11 (22.9)	12 (21.8)	23 (22.3)
10 (*n*, %)		16 (33.3)	15 (27.3)	31 (30.1)
Defecating (median [range])		10 [1–10]	10 [4–10]	10 [1–10]
0 (*n*, %)		0	0	0
1–4 (*n*, %)		9 (18.8)	1 (1.8)	10 (9.7)
5–9 (*n*, %)		12 (25.0)	9 (16.4)	21 (20.4)
10 (*n*, %)		27 (56.3)	45 (81.8)	72 (69.9)
Last 10 handwashes, used				
Water alone (*n*, %)		6 (12.5)	7 (12.7)	13 (12.6)
Water and bar soap (*n*, %)		42 (87.5)	48 (87.3)	90 (87.4)
Pregnancy planned (*n*, %)		23 (47.9)	30 (54.5)	53 (51.5)
Existing medical conditions				
Asthma (*n*, %)		2 (4.2)	0	2 (1.9)
Cardiac disease (*n*, %)		0	0	0
Coagulation disorder (*n*, %)		0	0	0
Congenital abnormalities (*n*, %)		0	0	0
Diabetes (type 2) (*n*, %)		1 (2.1)	0	1 (1.0)
High blood pressure (*n*, %)		0	0	0
Malaria (*n*, %)		6 (12.5)	14 (25.5)	20 (19.4)
Tuberculosis (*n*, %)		0	0	0
HIV				
0 (*n*, %)		32 (66.7)	39 (70.9)	71 (68.9)
1 (*n*, %)		7 (14.6)	8 (14.5)	15 (14.6)
2 (*n*, %)		9 (18.8)	8 (14.5)	17 (16.5)
STD (*n*, %)		1 (2.1)	0	1 (1.0)
Other (*n*, %)		0	0	0

Only half (47.9% (23/48) for control compared to 54.5% (30/55) for intervention) of the pregnancies were planned; however, all reported attending antenatal clinics. Most women, who reported unplanned pregnancies, wished to have had appropriate child spacing; though, they were not using family planning.

### Follow-up outcome

All, 100% (103/103), mothers in both clusters were followed up immediately after birth either in their homes or from health facilities. The majority of women had live births (94.5% (52/55) in intervention compared to 100% (49^1^/48) in control) and the research midwife completed the planned assessments for the immediate study visit (day 1 after childbirth) study visit. Three women in intervention clusters had stillbirths and did not have the immediate assessment performed.

At three months (90 days) after birth, most mothers with live babies were followed up and completed the final assessment (90.9%, 50/55) mother-baby pairs in intervention compared to (95.8%, 46/48) mothers with (95.9%, 47/49) live babies in control villages. This follow-up rate is commensurate with our predetermined acceptable follow-up criteria of above 80% both within 24 h and three months after birth. Of the seven women not followed-up on day 90, three had stillbirths, one had relocated to Kampala, one withdrew consent after childbirth, and two had neonatal deaths (Fig. 2 and Table 2).

**Table 2:** Birth outcome (mother)

		Control group	Intervention group	Difference (95% CI)
Sample size		48	55	103
Birth outcome				
Singleton (*n*, %)		47 (97.9)	55 (100.)	2.1 (not calculable)
Twin (*n*, %)		1 (2.1)	0	
Outcome at initial assessment				
Baby/babies survived (*n*, %)		47 (97.9)	51 (92.7)	− 5.2 (− 13.2:2.8)
Stillbirth (*n*, %)		0	3 (5.5)	
Baby died within 24 h (*n*, %)		1 (2.1)	0	–
Mother withdrew consent (*n*, %)		0	1 (1.8)	
Mother lost to follow-up after initial assessment (*n*, %)		0	1 (2.1)	–
Baby died after 10 days (*n*, %)		1 (2.1)	0	
Revised sample size for initial postnatal assessment		48*	51	
Mode of birth				
Spontaneous vaginal birth (*n*, %)		46 (95.8)	49 (96.1)	0.2 (− 7.5:8.0)
Caesarean section (*n*, %)		2 (4.2)	2 (3.9)	
Place of birth				
Home (*n*, %)		7 (14.6)	6 (11.8)	− 2.8 (− 16.2:10.5)
En route (*n*, %)		2 (4.2)	1 (2.0)	− 2.2 (− 9.0:4.6)
Health centre (*n*, %)		31 (64.6)	40 (78.4)	13.8 (− 3.8:31.5)
Hospital (*n*, %)		4 (8.3)	2 (3.9)	− 4.4 (− 13.9:5.0)
Other (*n*, %)		4 (8.3)	2 (3.9)	− 4.4 (− 13.9:5.0)
Person assisting birth (*n*, %)		48 (100.)	50 (98.0)	− 2.0 (not calculable)
Relative (*n*, %)		6 (12.5)	9 (17.6)	5.5 (− 8.7:19.7)
Traditional birth attendant (*n*, %)		1 (2.1)	3 (6.0)	3.9 (− 3.8:11.6)
Nursing assistant (*n*, %)		1 (2.1)	0	− 2.1 (not calculable)
Midwife/nurse (*n*, %)		38 (79.2)	42 (84.0)	4.8 (− 10.5:20.2)
Other (*n*, %)		5 (10.4)	2 (4.0)	− 6.4 (− 16.6:3.8)
Doctor		2	2	–
Neighbour		1	0	–
Retired midwife		1	0	–
Trained birth attendant		1	0	–
Sex of baby				
Male	(*n*, %)	21 (43.8)	22 (43.1)	− 0.6 (− 20.2:18.9)
Female	(*n*, %)	27 (56.3)	29 (56.9)	
Birthweight	Mean (s.d.)	3.3 (0.6) [2.0–4.5]^1^	3.4 (0.4) [2.8–4.6]^2^	0.11 (− 0.20:0.42) (*p* = 0.478)

^1^*n* = 25

^2^*n* = 24

*Mothers = 47, but as 1 mother had twin birth, babies = 48

### Birth outcomes

There were no major differences in clinical outcomes between both types of clusters for mothers and babies (Table 2). Spontaneous vaginal births were common in both types of clusters with only two caesarean sections in each arm. There were slightly more babies born at health facilities in the intervention arm (78.4%, 40/51) than the control arm (64.6%, 31/48), and the births were assisted by a local nurse/midwife (84.0% (42/51) intervention vs 79.2% (38/48) control) or doctors in case of caesarean sections (4.0% (2/51) intervention vs 10.4% (5/48) control). There were homebirths assisted by a relative, neighbour, while others were born en route to the facility or in the traditional birth attendants’ homes.

### Study protocol

Overall, the study protocol was feasible and acceptable; minor adjustments were made as the pilot progressed and errors were identified. The electronic data capture worked well, except for one participant whose initials changed during the follow-up visit which caused confusion with another participant. The research assistants believed that this error might have happened due to the small size of the handheld mobile smartphone (Samsung Galaxy S4). Three of the questions in the case report form had their text gradually revised to improve on context, which gradually improved data collection as the study progressed. The research assistants and those performing quality checks of the data (study coordinator, co-investigator, and data manager) all had no login errors onto the ODK smartphone system. Further, there were no extra logins required for synchronising data; the phone data automatically and easily synchronised whenever the phone connected to the internet.

### ABHR distribution

There were more women assigned to receive ABHR refills through the VHWs (70.9%; 39/55) than through the pharmacy (29.1%; 16/55), thus explaining the increased refills in that group (61.8% (34/55) compared to the pharmacy 21.8% (12/55)) (Table 3). Fifty women received a mean of two additional litres per participant (each received 1.1 l at the start). The mean number of refills was hence two with a mean volume of 3.1 l per participant. The women in both modes of distribution who did not return for a refill had stillbirths (three), had relocated (three), or had withdrawn from the study (one).

**Table 3:** ABHR distribution in intervention villages (*n* = 5) with participants (*N* = 55)

	Via VHW	Via pharmacy	Overall	Difference (95% CI)
Villages assigned	60.0% (3/5)	40.0% (2/5)	100% (5/5)	20 (−67.7:107.7)
Women wished to receive refills (self-reported)	70.9% (39/55)	29.1% (16/55)	100% (55/55)	41.8 (15.4:68.2)
Women actually refilled (self-reported at follow-up)	65.5% (36/55)^2^	25.5% (14/55)^3^	90.9% (50/55)	40 (12.4:67.6)
ABHR volume dispensed to women (litres; mean, range)	3.7 (2.8:4.1)	2.5 (2.0: 3.1)	3.1 (2.0: 4.1)	1.2 (1.09:1.31)
ABHR use/day (self-reported; mean, range)	7.0 (6.0:10)	6.2 (5.0:8.1)	6.6 (6.0:10.0)	0.8 (04.46:1.14)
ABHR accountability				
ABHR volume delivered (litres)	40	20	60	–
ABHR accounted for (litres)	40	15	55	–

^1^One village had one recruit, who had a stillbirth and did not do any ABHR refills

^2^Three mothers did not return for ABHR refills due to either stillbirth (1), study withdrawal (1), or relocation (1)

^3^Two mothers did not return for ABHR refills due to stillbirths (2)

VHWs were very systematic and accurately accounted for all the ABHR using the dispensing logs, compared to pharmacy workers who were unable to account for 5 l at the end of the study. Informal enquiry into the underlying reasons revealed that there were five midwives dispensing the ABHR at the pharmacy, and some midwives would dispense without completing the dispensing logs. Some pharmacy midwives also reported using the ABHR for their routine care, and some mothers reported being sent back from the pharmacy without a refill.

### ABHR use

The average frequency of ABHR use in the 5 intervention villages was 6.6 times per infant per day, but half stopped using it for at least 3 days continuously during the 3 months; most reported that they had simply forgotten to use it (Table 3).

### Risk of contamination (criteria: ABHR use in control sites less once a day)

There was no routine postnatal ABHR use reported in any of the control sites, except for one woman who was provided with a chlorhexidine gel from her local health facility. She had not applied it on the cord and was advised to use ABHR instead. A separate local study started towards the end of the BabyGel pilot. In this study, the health facility midwives provided chlorhexidine gel to every woman delivering in their facilities and advised mothers to apply the gel on the umbilical cord until it dropped off.

### Primary outcome

The a priori primary outcome was the rate of positive infection screen using the WHO’s IMCI screening criteria for infection (Table 4). The screening tool was positive for infection among a third of babies, i.e. 29.2% (14/48) in the intervention villages versus 31.4% (16/51) in the control villages.

**Table 4:** Infant infection outcomes

Primary outcome	Control group	Intervention group	Difference (95% CI)
	*n* = 48	*n* = 51	
Screen positive on IMCI form at any point within 90 days after birth	14 (29.2%)	16 (31.4%)	2.2 (− 15.9:20.3)
Sepsis deaths	1 (2.1%)	0	− 2.1 (not calculable)
Clinically diagnosed infection in health centre or hospital	8 (16.7%)	11 (21.6%)	4.9 (− 10.5:20.3)
Mother reported infant infection	19 (39.6%)	26 (51.0%)	11.4 (−8.1:30.9)
Infant received antibiotics	8 (16.7%)	14 (27.5%)	10.8 (− 5.4:26.9)
Infant was hospitalised	3 (6.3%)	6 (11.8%)	5.5 (− 5.7:16.7)
Total with any evidence of non-malaria infection (positive screen, maternal report, or clinical diagnosis)	27 (56.3%)	28 (54.9%)	− 1.3 (− 20.9:18.2)

VHWs completed the first four questions of IMCI screening tool with ease and accuracy (Fig. 4). However, the VHWs had challenges counting the baby’s respiratory rate or taking body temperature, both of which were recordable vital signs on the IMCI screening tool. They either left those questions blank (with no record entered) or filled a value that was wrong. Though they found challenges in taking temperature and counting respiratory rate and pulse rate, the VHWs made the judgement of high temperature through a touch of the baby with their palm and the fast breathing through experience or observation in the change of breathing from usual normal and completed the yes or no boxes. Further, the VHWs frequently did not attend to the mothers to complete the screening tool according to the proposed schedule in the protocol.

### Neonatal infection rate

Few babies actually had a clinical diagnosis of infection made in the study health centres or hospital (control 16.7% (8/48) compared to the intervention 21.6% (11/51); Table 4). The highest rates of identification of possible infant infection were through direct questioning of mothers. A half of women in the intervention arm (51.0%, 26/51) reported that their babies had suffered infections compared to two fifths in the control arm (39.6%, 19/48). Some babies received antibiotics from the local pharmacies and private clinics (control 16.7% (8/48) compared to intervention 27.5% (14/51)) but were neither screened nor reported to the study facilities. Overall, more than half (control 56.3% (27/48) compared to the intervention 54.9% (28/51)) of babies had some evidence of non-malaria infection either reported by mothers, evidenced in medical records, confirmed in the hospital, or screened positive on the WHO IMCI tool. There was no corresponding data collected centrally against which to compare. Only a half of all the sick babies had a positive IMCI screening tool (control 29.2% (14/48) compared to the intervention 31.4% (16/51)).

### Adverse events

There were no reported adverse reactions to the ABHR. There were, however, 15 severe adverse events (SAEs) unrelated to ABHR. These consisted of three stillbirths and one baby born with a cleft lip and palate (which was later repaired) in the intervention villages. In the control villages, there were two neonatal deaths: one occurred 16 h after birth due to asphyxia and another at 9 days after birth due to severe sepsis. The other SAEs were prolonged hospitalizations in both clusters.

### Verbal autopsy

There were five verbal autopsy assessments performed by research midwives before all investigators reviewed the completed assessment forms. One was an early neonatal death that occurred 16 h after birth, and the likely cause of death was asphyxia with no signs or symptoms of sepsis. However, in the late neonatal death that occurred 9 days after birth, the autopsy revealed the cause of death to be severe sepsis. The rest of the verbal autopsies were performed on the three stillbirths, and no cause of death was found.

### Intracluster correlation coefficients

The intracluster correlation coefficients (ICC) for possible infant infection in the first 90 postnatal days using the WHO IMCI screen were 0.238 in the control villages versus 0.171 in the intervention villages, and 0.171 for all clusters considered together. The corresponding values for the composite measure of infection (anyone with a positive screen, maternal report, or clinical diagnosis) were 0.179 in the control villages, 0.194 in the intervention villages, and 0.180 for both clusters together.

### The important changes to the methods after pilot trial commencement

The introduction of the expert children was an important change in the trial. These reinforced the mothers and other carers or visitors to use ABHR based on the three moments for hand hygiene poster. They further encouraged mothers to take their newborns to the clinics for immunisation.

Our planned follow-up schedule was that research team would only follow up participants on day 1 and day 90 after birth. However, the research team often visited the mothers outside the planned follow-up schedule, especially during recruitment of another eligible mother or follow-up of other mothers after birth for their scheduled dates of study appointment within the same or nearby village.

## Discussion

This pilot trial demonstrated that it was feasible to conduct a cluster randomised controlled trial of antenatal distribution of ABHR to prevent infections among newborns in the communities. The absence of any adverse reactions suggests that ABHR was safe to the newborns and communities. This pilot trial supports the conduct of a larger cluster randomised controlled trial in regard to cluster randomisation, village consent, screening, recruitment, individual informed consent rates, high fidelity, and acceptability of the ABHR intervention which was far above the set predetermined acceptable criteria of more than 80%. In this pilot trial, we demonstrated the relevance of home recruitment in promoting follow-up and retention of participants in community trials. Recruiting participants from their homes, with the GPS coordinates automatically captured onto the ODK system, made follow-up of participants simple even by another research assistant (who simply followed coordinates). There was no loss to follow-up.

The well-established network of village health team workers contributed to the ability to fully recruit pregnant women in this trial in each village. Only 176 pregnant women were required to be screened to fully recruit into this pilot trial, which was 1.7 times the enrolled sample. This translates into achievable recruitment rate for the large main cluster randomised controlled trial in the region.

We have demonstrated how the use of the VHWs for the distribution of ABHR refills worked efficiently. Community health workers have been demonstrated to be an important bridge to health care in other studies [46, 47]. However, pharmacy distribution was difficult with some mothers being refused ABHR and some ABHR missing from the pharmacies.

The study, further, confirms the efficient use of the ODK electronic data collection system in the field as seen in other African studies [48].

The reportedly high handwashing rate at baseline in both types of clusters could be explained by the behavioural changes following the study information, and possibly a cholera outbreak in the region that occurred prior the recruitment period and might have left a number of people informed about the need for handwashing.

The pilot trial showed that not all pregnancies end up into live births. Some ended up into stillbirths and neonatal deaths in these African settings in line with the existing literature [2, 9, 49]. In the planned main cRCT, considerations should be made to exclude stillbirths and neonatal deaths from further follow-up after death due to the sensitivity of their situation. Further, these women might benefit from bereavement support services. There is a value in supporting bereaved families through neonatal death and beyond [50, 51].

The training of mothers in the intervention clusters to use the ABHR based on the three moments for community neonatal hand hygiene [33] was a novel part of this pilot trial. It aimed at reinforcing the practice of hand rub for critical moments in the newborn’s life. Related training based on the WHO’s 5 Moments of Hand Hygiene has been reported to improve hand hygiene compliance in the health facilities [28]. Future research would include investigating the perspectives of women and baby carers towards these three moments of hand hygiene and whether this influences hand hygiene compliance in the homes. This pilot provided useful practical insights to explore further in the conduct of the main trial. One example is the creation of an ‘expert child’ within the home to improve adherence to the intervention. Though the expert children encouraged the use of the ABHR by anyone handling the baby and reminded the mother to take the baby to the health facility for immunisation and in the event of infant fever or any other concerns, there is not enough evidence in support of their effectiveness to promote home adherence to medical interventions. We, hence, would propose exploring this further in the main trial design and any other African community settings. Peer mentors and support groups have been found to improve adherence to HIV/AIDS interventions in the adult population [52–55], but it is not yet established whether the expert children would have a related benefit in a large community hand hygiene intervention.

The WHO IMCI screening tool was used in this pilot trial to describe the primary outcome of newborn infection. The pilot trial revealed difficulties with the IMCI screening tool as a measure of the primary outcome. It was difficult for the VHWs to accurately and completely fill the screening tool, with many of their screening forms having the wrong or missing vitals for temperature, pulse, and respiratory rate. Routinely, the VHWs are not routinely trained in measuring these vital signs and many have little formal education. In the future trial, therefore, we recommended that the VHWs complete as much of the screening tool as they could and refer any suspected sick babies directly to the facilities where the screening tool would be accurately completed by skilled health workers/midwives.

The screening tool also missed babies where the mother sought care through informal routes such as local pharmacies, drug shops, or private clinics with no designated staff for the infection screening process. In this economically deprived rural community, many women take their babies for care at local pharmacies and private clinics [56]. A reliance on the attendance of babies at the government clinics for the primary outcome, therefore, misses a number of infants with infection. For a future main trial, these difficulties could be avoided by carefully providing good training of frontline health workers in the use of the WHO IMCI criteria for the detection of infection in young infants [57]. In other settings, therefore, with better trained VHWs, the IMCI may still be an appropriate outcome.

This was a small pragmatic intervention study which is feasible to conduct, and its aim was not to cause lasting social change or implement a programme. However, there is some evidence that the trial intervention may have affected care-seeking behaviour. In the intervention group, there was a doubling of the number of women receiving antibiotics for their babies or being hospitalised, and more women reporting infant infection. Though this is in line with the theory of behaviour change framework [58], it could benefit exploring it further in the main trial. In this open study, where women are having daily contact with the intervention, it produces a daily reminder of the importance of reducing infection-related morbidity in the babies. This could reduce infection-related morbidity through preventative means (for example increasing the frequency of handwashing after toilet use) or by seeking earlier care for the infant at the first sign of any fever or any other symptom. However, it could also emphasise to women the danger of infection and lead to an increase in care-seeking behaviour and antibiotic use. It is critical therefore to use an objective outcome measure for all women. Use of the IMCI screen as an outcome would have provided this as it is independent of the stage of the illness and would have partially negated the effect of early care seeking. Alternatives would have been clinician-diagnosed or microbiologically proven infection. These would have had the same benefits but require massive resources.

Maternally reported infant antibiotic use may be an alternative primary outcome and has the benefit of picking up babies with infection, irrespective of where care was sought. Although it is likely to overestimate the rate of true infections (whether diagnosed by health care worker or microbiologically), it reflects a mixture of infection-related symptoms of the infants and maternal care-seeking behaviour. It is also a very important public health outcome given the actual and social cost of antibiotic use. However, a move to antibiotic use alone for the primary outcome measure would place the study at risk of ascertainment bias as women in the ABHR arm potentially seek care for their infants at an earlier stage. This bias could be eliminated through the use of a placebo hand rub in the control arm. This would allow a change of design to an individualised randomised trial, but there are obvious practical and ethical difficulties with the design and use of a placebo ABHR. Thus, we propose that, for the main trial, the primary outcome is a composite of infant infections in the first 90 days of life, defined as any one or more of (i) diarrhoea; (ii) lower respiratory tract infections; (iii) omphalitis; (iv) IMCI danger sign(s), all verified by health worker; (v) hospitalisation; or (vi) death. Each of the above composite items will also be secondary outcomes in their own right. These are meaningful clinical endpoints that are feasible to collect and are relatively unambiguous [59]. These outcome measures for the proposed main trial have been selected, to reflect the diversity of detection methods for sepsis as learned from the pilot trial. Each component of the primary outcome measure will be individually interrogated to ensure no illogical effects within the composite. Diarrhoea has long been regarded as a disease of poverty and is closely associated with poor hand hygiene of carers and unsanitary home environments. Lower respiratory tract infections are commonly spread to infants through droplets on hands (rather than by aerosol). Both are therefore preventable with good hand hygiene [4, 5, 60]. This would accelerate adoption and/or optimization of prevention products for poverty-related diseases in sub-Saharan Africa for use in pregnant women, newborns, and/or children.

Sample size in this trial was time-bound to accommodate the number of participants who would ably be followed up within the timelines of the 6 months.

The results of this pilot trial allow the sample size estimation for a future cluster randomised controlled trial comparing ABHR and usual care. The intracluster coefficient (ICC) estimate from this pilot trial was 0.17 (95% CI 0–0.65); however, a larger study reported ICC estimates from five similar context cluster randomised trials predominantly in the range 0.01 to 0.10 [61], which is considered more realistic than the pilot trial ICC. We hence propose to use this ICC instead of the pilot trial one for the future trial. To detect a reduction in the infection rate of ≥ 25% for control group rates down to 15% if ICC ≤ 0.01, or to detect a reduction in the infection rate of ≥ 33% for control group rates down to 5% if ICC ≤ 0.001 with a 90% power, 5932 participants are required. We have hence ignored multiple births for this calculation. This sample size should be achievable over a 2-year period of recruitment and across 72 villages. The sample size calculations are based on a primary endpoint of severe infection, with estimated rates of 5–30%.

### Limitations of the study

This pilot trial was limited in the estimate of potential ABHR effect due to the small sample size, as one would expect in a pilot and feasibility study [34, 62]. A larger cRCT is needed to draw conclusions about the effect of ABHR on the prevention of newborn infections in the community.

Although research midwives and clinicians objectively evaluated the newborns and mothers during the face-to-face encounters, we were only able to use self-reported assessments for a number of baseline variables and outcome measures. This included handwashing practices, ABHR acceptability, use of ABHR, and incidence of newborn infections from participants. This may have led to subjective bias in a number of ways. We assumed that women’s reports were correct and accurate and that women used the ABHR correctly instead of disposing it or using it for other purposes.

## Conclusion

This pilot trial results suggest that it is feasible to conduct a cluster randomised controlled trial (cRCT) of the provision of ABHR to postpartum mothers for the prevention of neonatal infection-related morbidity in the community in resource-poor settings. Our results indicate that home recruitment promotes excellent follow-up and retention of participants in community trials. This study informed the substantial changes necessary in the larger cRCT. Our study enabled us to calculate ICC for the sample size calculation of a future cRCT. We also recommend a change in the primary outcome of our study to a composite outcome considering multiple methods of infection detection in a larger cRCT.

The intervention was safe, and a large BabyGel cluster randomised controlled trial is now required.

## Abbreviations

ABHR: Alcohol-based hand rub
IMCI: Integrated Management of Childhood Illnesses
LNMP: Last normal menstruation period
MRC: Medical Research Council
ODK: Open Data Kit
SAE: Severe adverse event
UNCST: Uganda National Council for Science and Technology
VHW: Village health worker
WHO: World Health Organization
YICSSG: Young Infant Clinical Signs Study Group

## Footnotes

1 One mother had a pair of twins, hence more babies (49) than mothers (48).
